# Editorial: Diacylglycerol Kinase Signalling

**DOI:** 10.3389/fcell.2017.00084

**Published:** 2017-09-21

**Authors:** Isabel Merida, Andrea Graziani, Fumio Sakane

**Affiliations:** ^1^Department of Immunology and Oncology, CNB-CSIC Madrid, Spain; ^2^Department of Translational Medicine, University of Piemonte Orientale Novara, Italy; ^3^Division of Experimental Oncology, School of Medicine, Vita-Salute San Raffaele University Milan, Italy; ^4^Department of Chemistry, Graduate School of Science, Chiba University Chiba, Japan

**Keywords:** lipid signaling, immune system, synaptic transmission, immunotherapy of cancer, cytotoxic t cells, synaptic plasticity (LTP/LTD)

By gathering 10 reviews from leading scientists in a fast-evolving field, this Research Topic illustrates the contribution of diacylglycerol kinase (DGK) family members to the regulation of cell responses, and how their malfunction contributes to human pathologies.

DGK transform diacylglycerol (DG) into phosphatidic acid (PA). This simple reaction alters the levels of two lipids with central functions as second messengers, as phospholipid precursors, and as membrane modulators. The first DGK was purified and cloned by the Hideo Kanoh group in 1990; since then, the study of DGK regulation and functions has expanded and grows daily. Mammalian DGK comprise 10 isoforms that are particularly abundant in brain and hematopoietic organs. Not surprisingly, DAG effectors such as protein kinase C family members, mammalian Unc13 homologs, the chimaerin family of Rac GTPases, and the Ras GEF RasGRPs have critical functions in the regulation of nervous and immune synapses.

The mammalian DGK isoforms are grouped into five subtypes based on the presence of distinct regulatory domains. At least eight DGK isoforms are detected in the mammalian brain. In their review, Lee et al. summarize studies that link various DGK isoforms with certain features of synaptic plasticity. The generation of knockout mice for specific DGK isoforms has demonstrated their involvement in distinct aspects of brain function. These findings imply precise, isoform-specific control of lipid homeostasis that creates local environments suitable for triggering synaptic plasticity. Genome-wide association studies suggest relationships between specific DGK and human diseases that include bipolar disease and Parkinson's disease. Sakane et al. review these findings and discuss others such as mutations in the *DGKE* gene and its link to epilepsy and Huntington disease, or the more recent discovery of *DGKE* mutations associated with atypical hemolytic-uremic syndrome (aHUS).

The predominance of DGKε malfunction and disease might be related to this isoform's unique properties. DGKε is the only DGK family member that lacks regulatory domains, that is membrane-bound, and has substrate specificity for arachidonate-containing DG. Two articles in this series review DGKε properties and functions in depth, and cast new light on the links between DGKε defects and disease. Epand et al. describe the structural features that lend DGKε its arachidonoyl-DG specificity, its regulation by phosphorylation, and the identification of DGKε-interacting proteins. They discuss at length the unanticipated connection between recessive *DGKE* mutations and aHUS, recently uncovered by whole-exome sequencing. Although dysregulated complement activation is generally associated with all aHUS forms, the vast majority of aHUS patients that harbor *DGKE* mutations do not show this defect. DGKε is found at the endoplasmic reticulum (ER), a specialized site in membrane phospholipid synthesis and transport. In their review, Nakano et al. discuss the DGKε-specific contribution at the ER by generating arachidonoyl PA that feeds directly into the PI cycle. DGKε malfunction impairs PA generation and facilitates DG lipase-dependent DG degradation to 2-arachidonoyl glycerol, an endocannabinoid with important contributions to retrograde synaptic transmission.

All 10 mammalian DGK isoforms share a conserved catalytic domain and two protein kinase C conserved type I (C1) domains. The exception, with three C1 domains, is DGKθ, the lone member of the type V DGK class and the least-studied. In their detailed review, Tu-Sekine et al. cover DGKθ structure, enzymology and the latest results, which suggest its contribution to regulation of neurotransmission.

In addition to this notable role in brain, the DGK contribution to immune response control is another area of intensive research. T lymphocyte recognition of antigens presented on the antigen-presenting cell surface generates a polarized increase in membrane DG at the contact area. Sustained DG generation at the immune synapse facilitates activation of the Ras/ERK and PKC pathways, which ultimately dictate the fate of differentiated T cell populations. Chen et al. extensively describe the studies with DGKα- and ζ-deficient mice that have defined the contribution of these two isoforms to differentiation of CD8 T effector and memory populations, development of regulatory T cells (Treg), and lineage development of invariant natural killer cells. At difference from DGKα, expressed predominantly in T cells, DGKζ is expressed ubiquitously in hematopoietic cells. Singh and Kambayashi's review explores the immunomodulatory function of DGKζ in several cell types. The dual contribution of DGKζ, which can either potentiate or limit immune responses, adds new interest to its targeting. In addition to promoting defense against infection or cancer, DGKζ inhibition might have benefits by limiting immune responses in pathological conditions such as allergy and septic shock.

Due to their negative regulatory T cell function, DGKα and ζ have recently attracted interest as prospective targets, which might improve T cell-mediated tumor destruction. Two articles in this series examine the findings from their own and other groups that identify DGKα and ζ targeting potential in the fight against cancer. The contribution of DGKα as a cytosolic immune checkpoint is detailed reviewed in Noessner. On their review, Riese et al. consider DGKα and ζ contributions to limiting anti-tumor functions of engineered cytotoxic T cells. In both cases, pharmacological DGKα inhibitors induce recovery of cytotoxic functions by tumor-infiltrating cells, although the precise role of each isoform and the consequences of specific DGK ζ inhibition remain to be addressed. The continued search for new, more effective DGK inhibitors is a critical issue that is addressed by Sakane's group, who review the recent characterization of a new DGKα inhibitor.

DGK phosphorylation of DAG to PA is the principal pathway of DAG signaling termination. Through PA generation, DGK enzymes promote local alteration of membrane composition and electrostatic charge, which leads to recruitment and activation of additional sets of proteins. Baldanzi et al. illustrate how PA generation in specific membrane subdomains affects polarized responses such as immune synapse formation or cell migration.

We are deeply indebted to all the authors who have contributed to this Research Topic and to the dedicated reviewers who helped us achieve the highest quality standards. We gratefully acknowledge the valuable support of the Frontiers team in manuscript processing.

## Author contributions

IM wrote the editorial, AG and FS approved it.

### Conflict of interest statement

The authors declare that the research was conducted in the absence of any commercial or financial relationships that could be construed as a potential conflict of interest.

